# Evaluation of a static mixer as a new microfluidic method for liposome formulation

**DOI:** 10.3389/fbioe.2023.1229829

**Published:** 2023-08-22

**Authors:** Aoba Ota, Ayaka Mochizuki, Keitaro Sou, Shinji Takeoka

**Affiliations:** ^1^ Department of Life Science and Medical Bioscience, Graduate School of Advanced Science and Engineering, Waseda University, Tokyo, Japan; ^2^ Waseda Research Institute for Science and Engineering, Waseda University, Tokyo, Japan; ^3^ Institute for Advanced Research of Biosystem Dynamics, Waseda Research Institute for Science and Engineering, Waseda University, Tokyo, Japan

**Keywords:** liposome, encapsulation, microfluidics, static mixer, staggered herringbone micromixer, nanoparticle

## Abstract

**Introduction:** Microfluidic formulation of liposomes has been extensively studied as a potential replacement for batch methods, which struggle with problems in scalability and difficulty in modulating conditions. Although microfluidic devices are considered to be able to combat these issues, an adequate replacement method has yet to be established.

**Methods:** This paper examines the potential of a static mixer (SM) by comparing the encapsulation efficiency, loading, lamellarity, and user-friendliness with a commonly used microfluidic device, a staggered herringbone micromixer (SHM).

**Results:** In both devices, it was found that as the initial lipid concentration increased, the particle size increased; however, the overall particle size was seen to be significantly larger in the liposomes prepared with SM. PDI remained significantly smaller in SM, however, signifying that better control of the particle size was accomplished in SM. In addition, the encapsulation efficiency was slightly smaller in SM compared to SHM, and in both devices, the values increased as the initial lipid concentration increased. The increase in encapsulation efficiencies was significantly smaller than that of the theoretical encapsulation efficiency, and this was found to be due to the increase in lamellarity as the initial lipid concentration increased.

**Discussion:** In terms of user-friendliness, SM demonstrated significant advantages. The mixing elements could be taken out from the device, allowing for thorough cleaning of the element and device before and after experiments and ensuring experiments are conducted at virgin state in every round. Consequently, it was found that SM not only can produce uniformly distributed liposomes but has the potential to become a more practical method for liposome formulation with modifications in the mixing elements.

## 1 Introduction

Liposomes are biocompatible spherical nanocapsules formed with phospholipid bilayers, that have recently been extensively researched for their potential in medicine (Filipczak et al., 2020; Guimarães et al., 2021; Tenchov et al., 2021). Their amphipathic nature allows them to encapsulate both hydrophobic and hydrophilic molecules, and with their additional ability to be modified for targeted delivery, they are considered to have a high potential as a drug delivery system (Kraft et al., 2014; Bulbake et al., 2017; Liu et al., 2022). Much research has been conducted on liposomes which are beneficial in delivering hemoglobin (Takeoka et al., 1996; Sou et al., 2003), genes (Kikuchi et al., 1999; Obata et al., 2008), adenosine 5′-diphosphate (Okamura et al., 2010; Hagisawa et al., 2019), and contrast agents (Saito et al., 2005; Kostevšek et al., 2020). Encapsulation into liposomes has demonstrated benefits in targeting, controlled release (Bibi et al., 2012; Niu et al., 2012), stabilization, and protection from denaturation (Cortesi et al., 1996; Chaize et al., 2004).

Although *in vitro* experimentation of liposomes has shown substantial growth and would suggest great potential *in vivo*, their translation into the medical field and use clinically has not grown as expected. This has been observed to be due to problems in manufacturing, regulations, and the complexity of the technology (Sercombe et al., 2015). Manufacturing remains to be a great issue, with pharmaceutical industries struggling with reproducibility, scalability, and difficulty in adjusting the preparation conditions (Sercombe et al., 2015). The pre-existing methods that allow for successful liposome formulation are commonly batch methods such as ethanol injection method and thin-film hydration method (Šturm and Poklar Ulrih, 2021), and are not considered ideal in scalability, reproducibility, and modulation. Although batch methods are easily accessible to produce liposomes on an experimental scale in the laboratory, these methods only allow for the production of milliliter units, resulting in large amounts of waste when only necessary for *in vitro* experiments. In contrast, the scale would be rather small for preparing liposome samples for animal experiments. Such inefficiency in materials, costs, efforts, and time would be a big obstacle to the progress of liposome technology. On the other hand, in pharmaceutical manufacturing, at least kiloliter units of liposomes are required, so the batch method has to be conducted multiple times for an adequate amount to be manufactured. Additionally, adjusting the scale-up factors of the preparation conditions is complicated and requires proficient experience and skills in batch methods. For clinical uses such as when drugs are encapsulated into liposomes and delivered to tumors, accurate modification of liposome size is essential since it heavily impacts its dynamics (Nagayasu et al., 1999). However, batch methods are incapable of preparing a monodisperse sample of liposomes, and often require multi-step processing such as extrusion to unify and control the size of the liposomes (Šturm and Poklar Ulrih, 2021). Since extrusion requires the liposomes to pass through membrane filters of decreasing pore sizes, clogging of the membrane filters and loss of the lipids and encapsulated molecules have been serious problems in this step.

Microfluidic formulation of liposomes can combat these issues, allowing for a more uniformly distributed size of liposomes to be generated in a continuous flow-synthesis method (Jahn et al., 2008). T- and Y-shaped micromixers allow for two liquids to consecutively be mixed, allowing there to be no limit to how much liposomes are prepared as long as liquids are being passed through; in other words, preparation of minuscule volumes to potentially an endless supply can be made possible without changing the conditions. In addition, in a microfluidic device, by simply altering different parameters such as the total flow rate (TFR, the volume at which the liquids are passed through the device per minute), flow rate ratio (FRR, the ratio at which the organic and aqueous solution are pushed through the device), and the initial lipid concentration of the organic solution (ILC), properties of the resultant sample can be modulated. Thus, there is no need for subsequent size control steps such as extrusion, minimizing the hassle and loss during liposome preparation.

A staggered herringbone micromixer (SHM) is a microfluidic device that incorporates a series of asymmetric protrusions in a flow channel for rapid mixing. To this day, the SHM device has been evaluated to produce lipid-based nanoparticles including liposomes and lipid nanoparticles (Chen et al., 2012; Cheung and Al-Jamal, 2019). By modulating the preparation conditions, SHM can prepare nanoparticles that have controlled size and can efficiently encapsulate therapeutic agents such as hydrophobic drugs and mRNA (Chen et al., 2012; Kastner et al., 2015). While microfluidic technologies have many advantages, the devices used in the experiments are problematic in that blockages frequently occur, and the devices are extremely fragile to exterior force. Many of these chips have channels that are micrometers in width and thickness, resulting in the devices being clogged with the smallest dust particles finding their way through the channel. Therefore, this method does not seem promising especially if macromolecules or molecules with high aggregation are intended to be encapsulated. Solvent resistance is another limitation of these devices which are fabricated by soft lithography. The performance of devices made of polydimethylsiloxane, which is a commonly used material for conventional microfluidic devices, may change by swelling and deformation when in contact with organic solvents. Furthermore, the low volumetric throughput of the microfluidic device due to the narrow channels is another disadvantage for the translation of laboratory-scale preparation to large-scale production for clinical study and commercial scale.

**FIGURE 1:**
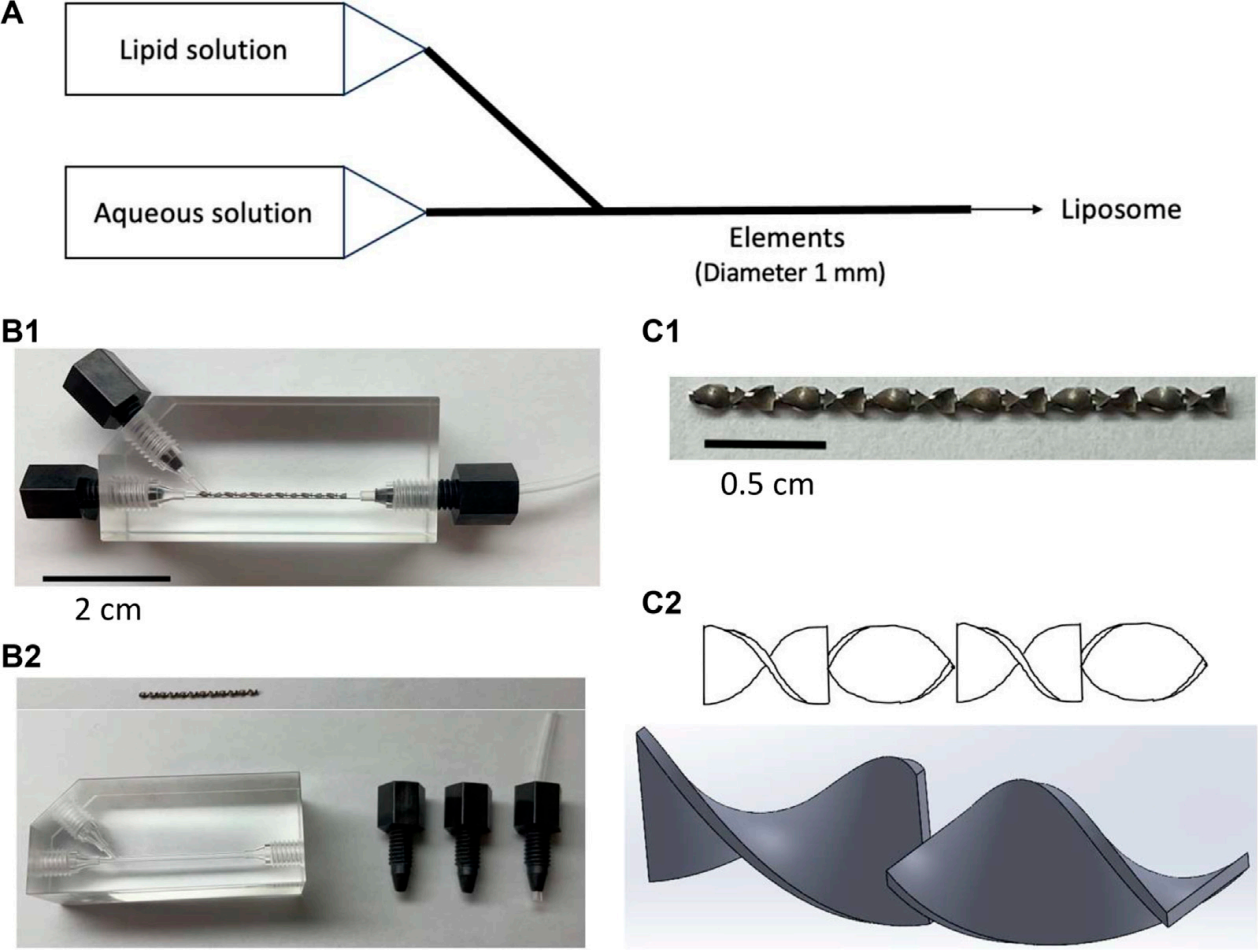
Pictures and schemes of the static mixer. **(A)** Schematic of a microfluidic method for liposome preparation using a static mixer with an element in a channel of 1 mm diameter, **(B1)** static mixer assembled, **(B2)** disassembled static mixer with the element removed and placed above the device, **(C1)** zoomed-up image of the element, and **(C2)** scheme of the elements viewed from various angles.

A static mixer (SM), a device that can mix two liquids in a channel of a few millimeters in diameter with a structure shown in Figure 1, is considered to have the potential for liposome preparation. SMs are motionless mixers that have been applied in the blending of fluids, solids gases, and heat transfer (Thakur et al., 2003). Their structure allows for thorough homogenization and due to mixing being conducted without agitation, they are low in energy consumption and require less maintenance, resulting in their prevalence in the pharmaceutical industry, food engineering, and petrochemical industries (Thakur et al., 2003). SMs typically have a larger channel than a microfluidic device, which allows them to be less prone to blockages and allows for high volumetric throughput.

This work utilized a SM which has a channel of 1 mm diameter, allowing for the observation of blockages with the naked eye. Furthermore, the structure of the device allowed for the elements in the channel to be taken out and washed. This ensured that the device was completely cleansed after each experiment, allowing all experiments to be conducted at virgin state. With the aforementioned user-friendliness being given, this work aims to further understand the potential of using SM in liposome preparation and also evaluate the differences it may hold when compared to the conventional microfluidic device. In this paper, the liposomes produced in SHM are compared to that of SM and evaluated to consider the potential of a static mixer in a liposome preparation.

## 2 Materials and methods

### 2.1 Materials

For lipid components, 1, 2-dipalmitoyl-*sn*-glycerol-3-phosphocholine (DPPC) and cholesterol were purchased from Tokyo Chemical Industry Co. Ltd. (Tokyo, Japan), 1, 5-dihexadecyl-*N*-succinyl-L-glutamate (DHSG) was purchased from Nippon Fine Chemical Co. Ltd. (Osaka, Japan), and 1, 2-distearoyl-*sn*-glycero-3-phosphoethanolamine-*N*-[monomethoxy poly (ethylene glycol) (5000)] (PEG-DSPE) was purchased from NOF Co. Ltd. (Tokyo, Japan). For fluorescent dyes, calcein was purchased from Dojindo Laboratories (Kumamoto, Japan), while 1,1′-dioctadecyl-3,3,3′,3′-tetramethylindodicarbocyanine, 4-chlorobenzenesulfonate salt (DiD) was purchased from Thermo Fisher Scientific (Waltham, United States).

### 2.2 Liposome formulation with SM and SHM

Liposomes were formulated by using a static mixer (SM) and a staggered herringbone micromixer (SHM) under comparable conditions to understand the potential of SM in liposome formulation when compared to an established microfluidic method such as SHM (Cheung and Al-Jamal, 2019). A modified K-series SM was provided from Noritake (Aichi, Japan) (Figure 1), while SHM (Darwin microfluidics, 2023) was purchased from Darwin Microfluidics (Paris, France) (Supplementary Figure S1). DPPC, cholesterol, DHSG, and PEG-DSPE were dissolved in t-butyl alcohol at a molar ratio of 5:5:1:0.066 and DiD was then further added at 0.2 mol% to the total lipid. The mixed solution was freeze-dried to obtain a mixed lipid powder for stock. An aliquot of the freeze-dried powder was dissolved in filtered 99.5% ethanol (Fujifilm Wako Pure Chemical, Japan) at concentrations of 15–90 mg/mL before each round of the experiment to prepare the lipid solution. Calcein was applied for evaluating the encapsulation efficiency of liposomes. Calcein is widely used as a water-soluble fluorescent marker to evaluate the encapsulation capacity and release properties of liposomes because calcein can be stably encapsulated into liposomes (Grit et al., 1992; Berger et al., 2001). Also, the encapsulation efficiency of calcein into liposomes prepared by a filter extrusion method well agrees with the theoretical value calculated for unilamellar liposomes in the assumption that there is no interaction between the lipid bilayer membrane and encapsulated molecules (Xu et al., 2012). Calcein was dissolved in PBS (Takara Bio, Japan) using NaOH (final concentration 3 mM) to prepare a 1 mM calcein solution as the aqueous solution. The calcein solution was passed through a hydrophilic filter with a pore size of 0.22 µm (Merck Millipore, United States) after it had fully dissolved. Both solutions were filtered to ensure that no blockages would be caused by foreign particles. The mixed lipid ethanol solution and aqueous calcein solution were passed through the devices (SM and SHM) at TFR of 1,500, 2000, and 2,500 μL/min and aqueous-to-ethanol FRR of 3,4, and 5. The TFR and FRR were adjusted by a syringe pump (Legato 111, KD Scientific, United States). In order to accurately collect liposomes that were generated with the set TFR and FRR, the liposomes produced in the beginning and towards the end of the flows were not collected. The lipid-in-ethanol concentrations were set as 15, 30, 45, 60, and 90 mg/mL, presented as initial lipid concentration (ILC).

### 2.3 Liposome purification

The resultant liposomes were purified by ultrafiltration to remove ethanol and calcein that were not encapsulated into the liposomes. The centrifugal ultrafiltration device Vivaspin^®^6, with membrane 100,000 MWCO (Sartorius, Germany) was used, allowing for liposomes to remain in the concentrator while the outer aqueous layer with the unencapsulated calcein and ethanol was dropped into the filtrate container. The sample was centrifuged until the outer aqueous layer was diluted 1,000 times with PBS, and the resultant liquid on the top was collected with a pipette with the liposomes on the membrane being washed down and collected with PBS.

### 2.4 Characterization and encapsulation properties of liposomes

Particle size, polydispersity index (PDI), lipid recovery rate (LRR), encapsulation efficiency (EE), and loading were measured for analysis. Particle size and PDI were measured by Zetasizer Nano (Malvern Panalytical, United Kingdom). LRR, loading, and EE were calculated from equations 1, 2, and 3, respectively. An aliquot of the obtained liposome dispersion (100 µL) was mixed with ethanol (900 µL) to solubilize the liposomes, and the lipid concentration was calculated from the DiD fluorescence intensity in the ethanol solution (λ_ex_ = 635 nm, λ_em_ = 670 nm). The calcein concentration was calculated from the fluorescence intensity measured after solubilizing the liposomes with octyl glucoside at a final concentration of 45 mM in PBS (λ_ex_ = 480 nm, λ_em_ = 530 nm). All fluorescence intensities were measured using the SynergyH1 fluorescent plate reader (BioTek, United States). In the following equations, mixed solution (MS) refers to the aqueous solution and lipid solution mixed by pipetting in a defined mixing ratio without having passed through any device.

$$LRR\;\left[\%\right]=\frac{Lipid\;concentration\;of\;sample\;after\;passing\;through\;device\;\left(\frac{mg}{mL}\right)}{Lipid\;concentration\;of\;MS\;\left(\frac{mg}{mL}\right)}\times 100$$ (1)

$$Loading=\frac{Calcein\;concentration\;of\;sample\;after\;purification\;\left(mM\right)}{Lipid\;concentration\;of\;sample\;after\;purification\;\left(\frac{mg}{mL}\right)}$$ (2)

$$EE\;\left[\%\right]=Loading\times \frac{Lipid\;concentration\;of\;sample\;after\;passing\;through\;device\;\left(\frac{mg}{mL}\right)}{Calcein\;concentration\;of\;MS\;\left(mM\right)}\times 100$$ (3)

The theoretical encapsulation efficiency (TEE) was calculated using a mathematical model created by Xu et al. (Eq. 4), which predicts the encapsulation efficiency in unilamellar liposomes (Xu et al., 2012).

$$TEE\;\left[\%\right]=\frac{\sum_{i}\left(\left(\frac{4}{3}\right)\pi {\left(r_{i}-d\right)}^{3}∙\left(c∙V∙N_{A}\right)/\sum_{i}\left(4\pi \left[r_{i}^{2}+{\left(r_{i}-d\right)}^{2}\right]∙\frac{P_{i}}{a}\right)∙P_{i}\right)}{V}\times 100$$ (4)

In the above equation, d , $r_{i}$ , $P_{i}$ , c , *a* and V refers to the membrane thickness of DPPC bilayer membrane (4.8 nm) (Lewis and Engelman, 1983), the radius of the liposome i , probability of $r_{i}$ , lipid molar concentration, known average molecular area of lipid in monolayer membrane of DPPC and cholesterol at a molar ratio of 1:1 (0.43 nm^2^) (Kodama et al., 2004), and total sample volume, respectively. The c and V were assigned as lipid concentration of a liposome dispersion calculated from FRR and ILC (mM), and the volume of liposome dispersion (mL), respectively. *N* *_A_* is Avogadro’s number which is equal to 6.02 × 10^23^. The variables $r_{i}$ and $P_{i}$ are the particle size radius measured by the Zetasizer and the probability of the presence of vesicles of the entered size, respectively. To calculate $P_{i}$ , $r_{i}$ and size distribution were substituted. However, this model assumes that the liposomes are spherical, have an inner aqueous phase entrapped with a single lipid bilayer separated from the outer aqueous phase, the encapsulated molecule is hydrophilic and has the same concentration as the outer aqueous phase, and the particle size follows a Log-Normal distribution (Xu et al., 2012).

For comparison of the lamellarity of liposomes, a fluorescence probe, 6-*p*-toluidino-2-naphthalenesulfonic acid (TNS) (Abcam, United Kingdom) was used to compare the total surface area of liposomes, in which the ratio of the surface area at the same lipid concentration represents the number of bilayer membranes (Takeoka et al., 1996). For the liposomes prepared in the SHM and SM, TFR 1500 μL/min, FRR 3, and ILC 15, 45, and 90 mg/mL were used (six preparation conditions in total). A liposome sample with the same lipid composition was prepared using a probe-type sonicator (Sonifer 250, Branson), and this was defined as a standard of unilamellar liposomes. The lipid concentration in the liposome samples was determined using a phospholipid assay kit (Fujifilm Wako Pure Chemical, Japan), and the samples were diluted to have the same concentrations. TNS was added to all liposome samples with a five-step gradient in lipid concentration and incubated at ambient temperature for 12 h before fluorescence was measured (λ_ex_ = 321 nm, λ_em_ = 400 nm). After the fluorescence measurement, the relationship between the amount of liposome sample added and the fluorescence intensity was plotted on a graph, and the slopes were calculated. The number of the bilayer membranes of liposomes (lamellarity) was calculated by dividing the slope of the standard unilamellar liposomes by the slope of the liposome samples, with the assumption that the number of the lipids composing each lipid layer is the same as the number of the lipids composing the outermost layer of liposomes.

### 2.5 Scatterplot matrix

A scatterplot matrix was performed to examine linear correlations between multiple variables. Scatterplot matrix analysis was conducted using Microsoft Excel for Mac version 16.73. The relationship between the parameters and the characteristics of the liposomes could be visualized by the scatterplot matrix. The *p*-value was calculated from the t-value, which was calculated from the Pearson correlation coefficient and the number of plots using Microsoft Excel for Microsoft 365 MSO version 2307. Statistical significance was considered at *p* < 0.05.

## 3 Results

### 3.1 SHM device

**FIGURE 2:**
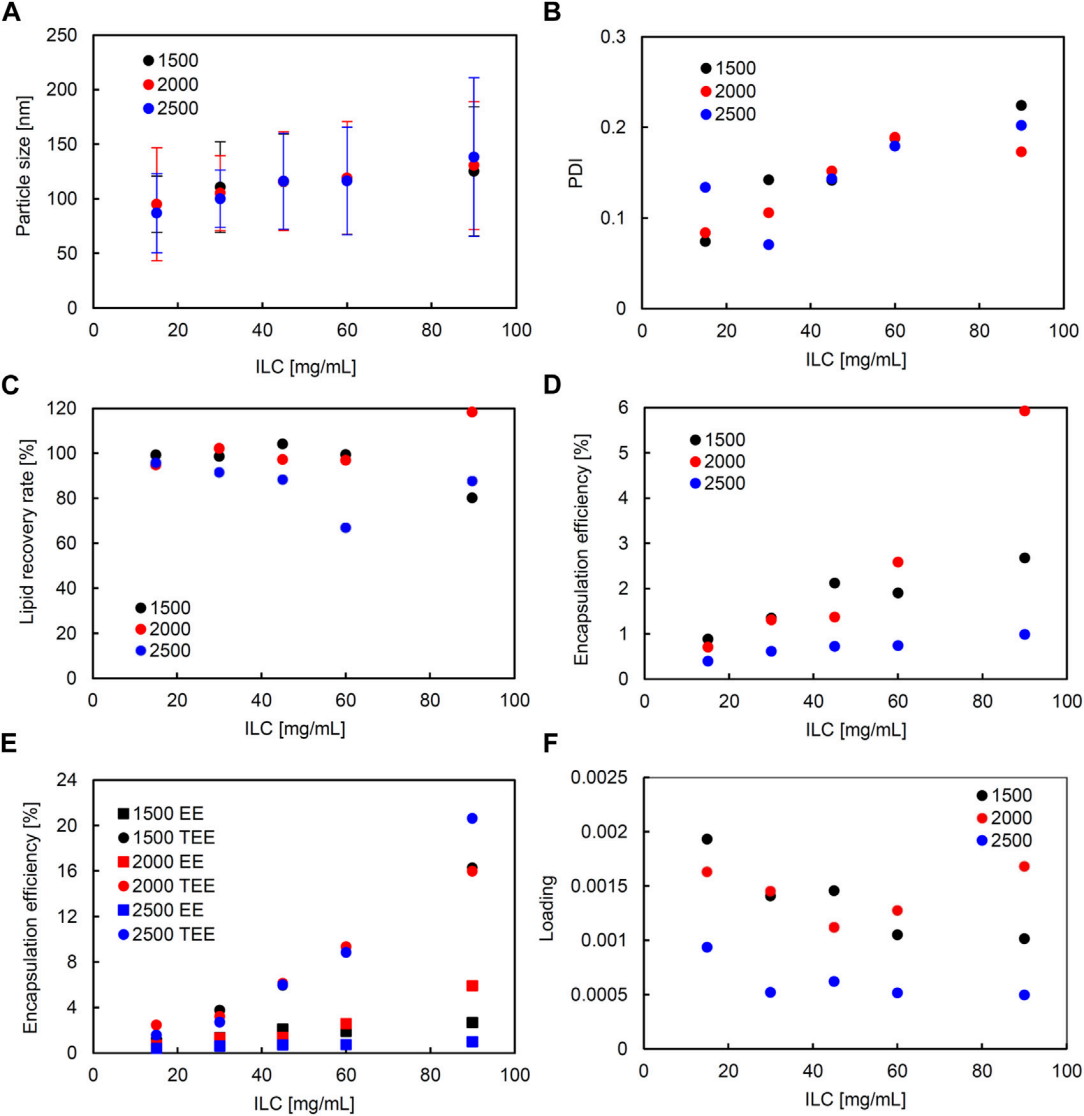
Comparison of liposome characteristics generated under total flow rate (TFR) of 1,500, 2000, and 2,500 μL/min in a staggered herringbone micromixer (SHM) device. The flow rate ratio (FRR) was fixed at 3. **(A)** Particle size, **(B)** polydispersity index (PDI), **(C)** lipid recovery rate (LRR), **(D)** encapsulation efficiency (EE), **(E)** theoretical encapsulation efficiency (TEE) and EE, and **(F)** loading.

**FIGURE 3:**
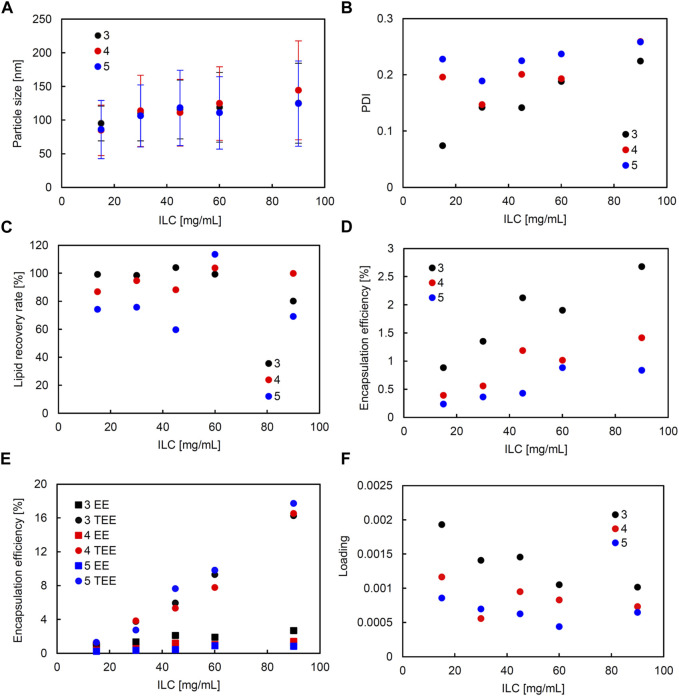
Comparison of liposome characteristics generated under flow rate ratio (FRR) of 3, 4, and 5 in a staggered herringbone micromixer (SHM) device. The total flow rate (TFR) was fixed at 1,500 μL/min. **(A)** Particle size, **(B)** polydispersity index (PDI), **(C)** lipid recovery rate (LRR), **(D)** encapsulation efficiency (EE), **(E)** theoretical encapsulation efficiency (TEE) and EE, and **(F)** loading.

In the liposomes formulated in SHM, correlation coefficients of higher than 0.5 were observed between ILC and particle size, ILC and PDI, particle size and PDI, ILC and EE, and particle size and EE (Supplementary Figure S2). Figure 2 shows the characteristics of the lipid particles obtained using SHM when the FRR was fixed at 3 and the TFR was set to 1,500, 2000, or 2,500 μL/min. Figure 3 shows the characteristics of the lipid particles when the TFR was fixed at 1,500 μL/min and the FRR was set to 3, 4, or 5. ILC was varied from 15 to 90 mg/mL at each condition. The mean particle size of the obtained liposomes was between 86–144 nm with a unimodal size distribution (Figures 2A, 3A). The particle size increased as the ILC was increased at any combinations of TFR and FRR, and the average size was seen to range from 91 nm at ILC 15 mg/mL to 131 nm at ILC 90 mg/mL. In addition, as the ILC and particle size increased, the particle size distribution was also seen to increase.

The obtained liposomes exhibit PDI values of 0.071–0.259 (Figures 2B, 3B). The correlation coefficient between the ILC and PDI was 0.64, with the PDI increasing as the ILC increased (Supplementary Figure S2). This characteristic was seen to be the most evident in Figures 2B, 3B at TFR 1500 μL/min, FRR 3 where the PDI was 0.074 at ILC 15 mg/mL and 0.224 at 90 mg/mL. The values of calculated LRR were in the range of 60%–118% without a clear correlation with other variables (Figures 2C, 3C; Supplementary Figure S2).

The correlation coefficient was 0.57 between the ILC and EE (Supplementary Figure S2) with the EE increasing as the ILC was increased under any combination of FRR and TFR (Figures 2D, 3D; Supplementary Figure S2). Furthermore, it was also found that EE was higher at lower FRR in all ILCs (Figure 2D). A distinct difference could be observed at ILC 90 mg/mL, where the EE was 2.7%, 1.4%, and 0.8% for FRR 3, 4, and 5, respectively.

The particle size and EE was seen to have a correlation coefficient of 0.50 with the EE increasing as the particle size got larger (Supplementary Figure S2). When looking at the data for TFR 2000 μL/min, it can be seen that at ILC 15 mg/mL when the particle size was 95 nm, the EE was 0.7%; whereas, at ILC 90 mg/mL, when the particle size was 131 nm, the EE was 5.9%, increasing the EE 8-fold with the increase in particle size. In addition, it was found that in all conditions, TEE also increased when ILC increased, with the gap between TEE and EE expanding proportionally to ILC (Figures 2E, 3E; Supplementary Figure S4). The loading varied between 0.000439 and 0.00193, with all values obtained at TFR 2500 μL/min lower than 0.001 (Figures 2F, 3F).

### 3.2 Static mixer

**FIGURE 4:**
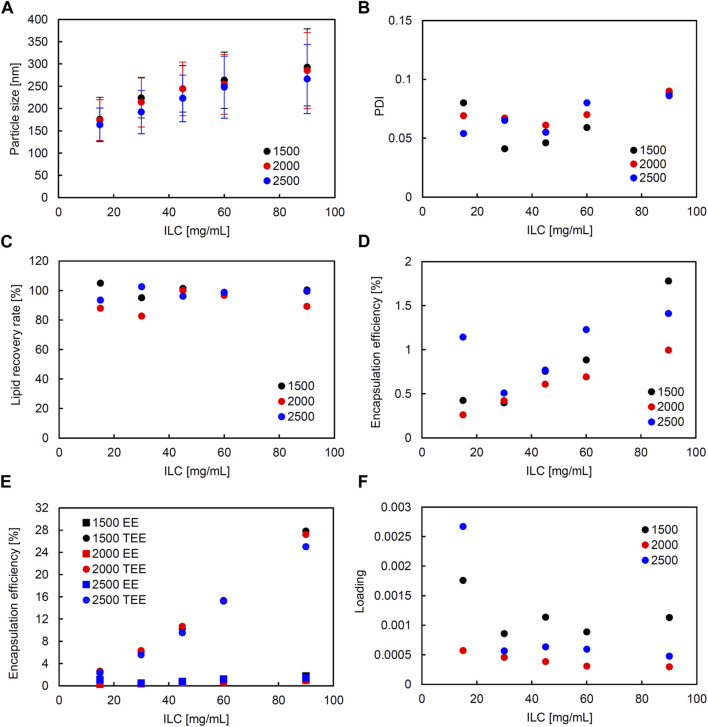
Comparison of liposome characteristics generated under total flow rate (TFR) of 1,500, 2000, and 2,500 μL/min in a static mixer. The flow rate ratio (FRR) was fixed at 3. **(A)** Particle size, **(B)** polydispersity index (PDI), **(C)** lipid recovery rate (LRR), **(D)** encapsulation efficiency (EE), **(E)** theoretical encapsulation efficiency (TEE) and EE, and **(F)** loading.

**FIGURE 5:**
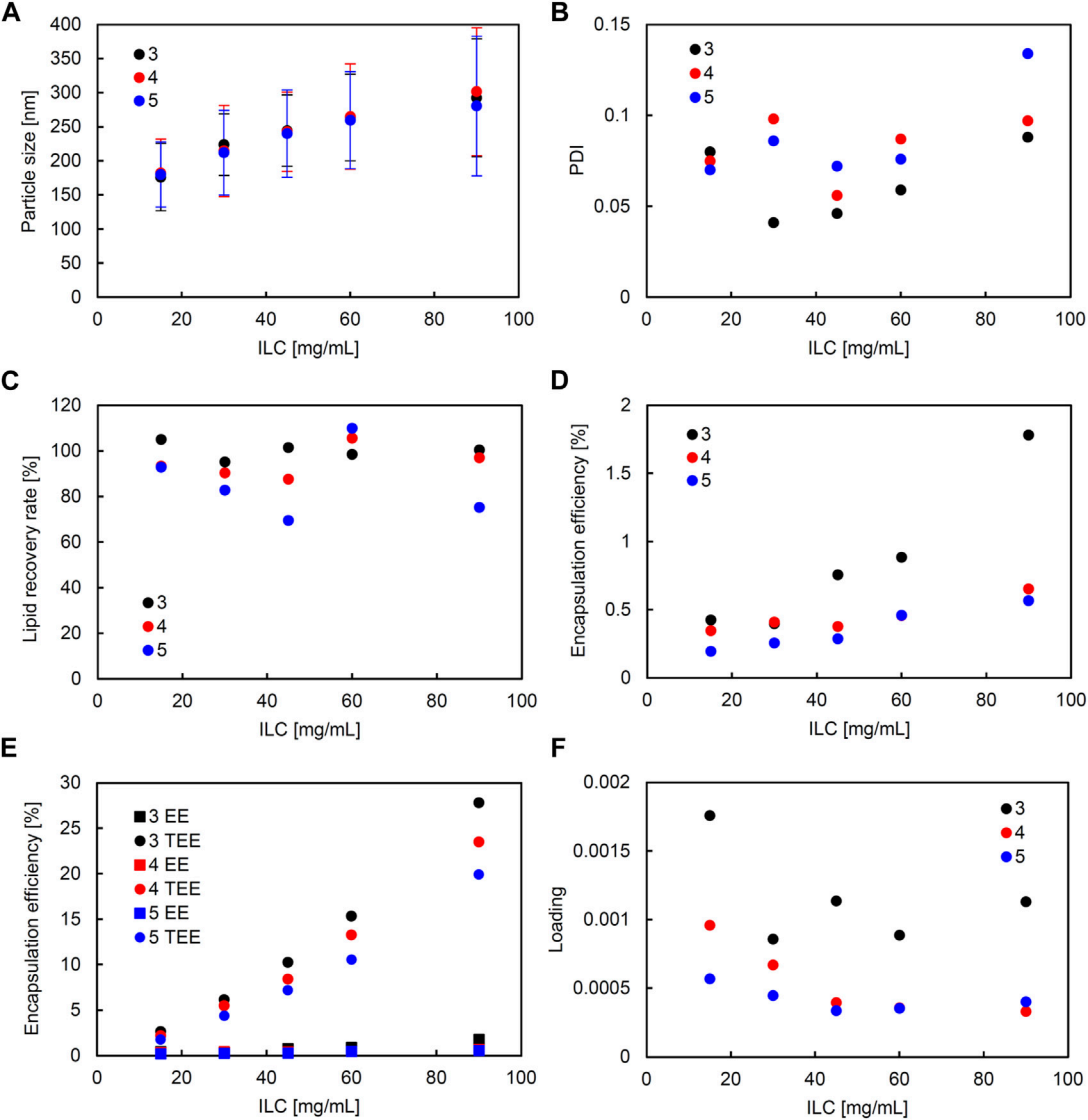
Comparison of liposome characteristics generated under flow rate ratio (FRR) of 3, 4, and 5 in a static mixer. The total flow rate (TFR) was fixed at 1,500 μL/min. **(A)** Particle size, **(B)** polydispersity index (PDI), **(C)** lipid recovery rate (LRR), **(D)** encapsulation efficiency (EE), **(E)** theoretical encapsulation efficiency (TEE) and EE, and **(F)** loading.

Similarly, in the SM, correlation coefficients with significance (higher than 0.5 and smaller than −0.5) were seen between the following: ILC and particle size, ILC and EE, and particle size and loading (Supplementary Figure S3). Figure 4 shows the characteristics of the liposomes obtained using SM when the FRR was fixed at 3 and the TFR was set to 1,500, 2000, or 2,500 μL/min. Figure 5 shows the characteristics of the lipid particles when the TFR was fixed at 1,500 μL/min and the FRR was set to 3, 4, and 5. ILC was varied from 15 to 90 mg/mL at each condition. The particle size was seen to increase as ILC was increased, with a correlation coefficient of 0.96 (Supplementary Figure S3). This was evident at TFR 2500 μL/min where the particle size increased from 164 nm at ILC 15 mg/mL to 304 nm at ILC 90 mg/mL. One thing to note was that although the particle size drastically increased, the PDI mostly remained consistently under 0.1 (Figures 4B, 5B). Furthermore, as can be seen in Figure 4A, as TFR was increased, the particle size was seen to decrease. The largest difference was seen at 30 mg/mL where the particle size was 224 nm at TFR 1500 μL/min and 192 nm at TFR 2500 μL/min. In all conditions, as can be seen in Figures 4C, 5C, the LRR mostly remained within the range of 70%–120%.

A correlation coefficient of 0.58 was seen between ILC and EE (Supplementary Figure S3). In all conditions, as ILC was increased, EE was also seen to increase (Figures 4D, 5D). The most drastic increase was seen in the condition of TFR 1500 μL/min, FRR 3 where EE was 0.4% at ILC 15 mg/mL and 1.8% at ILC 90 mg/mL, an increase of over 4-fold (Figure 4D).

The particle size and loading had a barely significant correlation of coefficient −0.50, where, as the size increased, the loading decreased. This is evident in Figures 4A, 5A; Figures 4F, 5F, where the particle size increased and loading decreased as ILC was increased. In addition, as can be found from Figures 4E, 5E, in all conditions, the gap between the TEE and EE increased as ILC was increased (Supplementary Figure S4).

### 3.3 SHM and SM comparison

**FIGURE 6:**
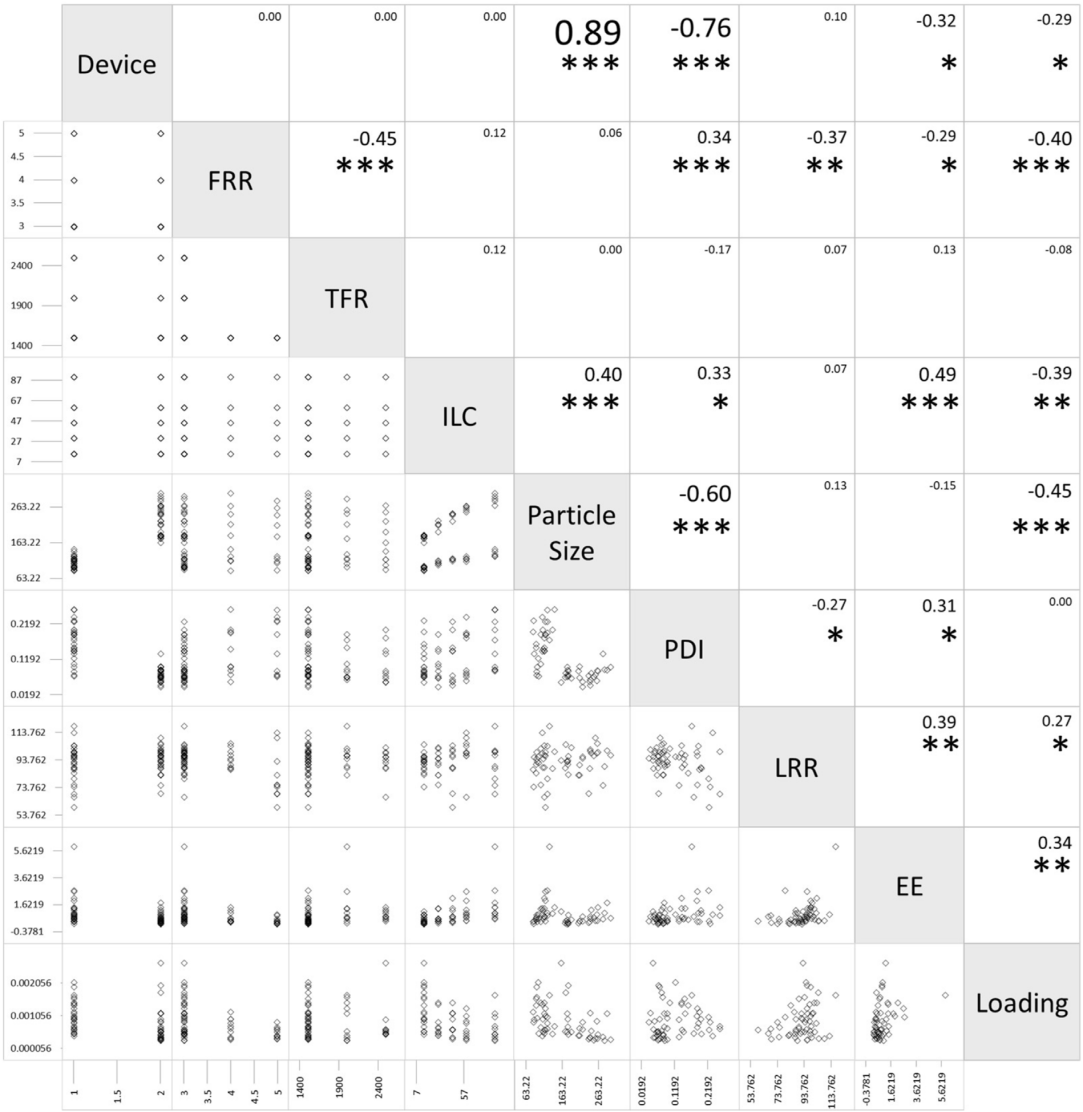
Scatterplot matrix of flow rate ratio (FRR), total flow rate (TFR), initial lipid concentration (ILC), particle size, polydispersity index (PDI), lipid recovery rate (LRR), encapsulation efficiency (EE), and loading for the static mixer and staggered herringbone micromixer (SHM) device (Device 1 as the SHM device, Device 2 as the static mixer). The values in the boxes represent the Pearson correlation coefficients between each pair of variables. Statistical significance of the Pearson correlation coefficient is represented as *p < 0.05, **p < 0.01, and ***p < 0.001.

When the two devices were compared in the scatterplot matrix, it was found that the difference in device yielded a difference in particle size and PDI with correlation coefficients of 0.89 and −0.76, respectively (Figure 6). The particle size was significantly smaller in all ILCs for the SHM device when compared to the SM. The average size difference between the liposomes generated using the two devices was 122 nm when compared under the same conditions. The size difference was seen to increase as ILC was increased, with the difference being 85 nm, 106 nm, 124 nm, 141 nm, and 155 nm, for 15, 30, 45, 60, and 90 mg/mL, respectively.

A correlation coefficient of −0.76 was seen between the device and the PDI with SHM having a much larger PDI than SM. The average PDI size for SHM and SM were 0.170 and 0.073, respectively, with the PDI being 2.3 times larger for SHM.

Furthermore, from the scatterplot matrix, it was found that the particle size and PDI had a correlation coefficient of −0.60. Figure 6 shows a distinct difference between the two particle size ranges and the PDI. The smaller particle sizes are liposomes formulated by SHM and a large range of PDI can be seen; whereas, in the larger particle sizes of liposomes formulated with SM, the PDI remains small. This can also be seen in Figures 2B, 3B; Figures 4B, 5B, where the PDI is seen to range up until approximately 0.26 for SHM, while SM was, excluding one condition, able to maintain the PDI at under 0.1.

TNS fluorescence was measured for six liposome samples formulated at ILC 15, 45, and 90 mg/mL, each with an SHM and SM. For the measurement, the lipid concentration of liposomes was unified, based on the results of a phospholipid assay. The slopes which reflect the relative value of the total surface area of liposomes were found to be 9.3, 7.5, and 6.6 for SHM, and 5.1, 1.7, and 0.73 for SM at ILC 15, 45, 90 mg/mL, respectively (Supplementary Figure S5A). The standard for unilamellar liposomes made through hydration and probe sonication had a slope of 8.6. From this, the lamellarity of the liposomes was calculated to be 0.93, 1.2, and 1.3, for the SHM, and 1.7, 5.1, and 11.9 for SM at ILC 15, 45, and 90 mg/mL, respectively (Supplementary Figure S5B). Thus, it was found that liposomes formed in SM at ILC 45 and 90 mg/mL are more multilamellar or multivesicular, with the lamellarity increasing proportionally to the ILC (Supplementary Figure S5B). When the appearance of liposome dispersions formulated without fluorescent dyes was compared, it could be seen that the SHM samples are seen to be less turbid than the SM samples (Supplementary Figure S6). Furthermore, the differences in turbidity between SM and SHM samples were more distinct as the ILC got higher.

## 4 Discussion

In both SHM and SM, the ILC had a great impact on the particle size with a positive correlation. A much greater increase in particle size was seen for SM, where the particle size at 90 mg/mL was seen to increase an average of 111 nm from 175 nm at 15 mg/mL; whereas, for SHM, the particle size at 90 mg/mL was only seen to increase an average of 41 nm from 91 nm at 15 mg/mL. Moreover, the overall particle size was significantly smaller for SHM compared to SM. However, PDI remained significantly smaller in SM than in SHM, indicating that the liposome samples formulated in SM are more uniformly distributed. While the PDI was seen to be high for SHM as ILC was increased, SM was mostly able to maintain PDI under 0.1. This shows that SM is more able to mix the solutions equally with little unevenness in mixing. Since the accurate manipulation of particle size is essential in medicinal applications, small PDI in SM is highly promising for future applications.

In addition to the ILC contributing to the particle size, TFR is seen to have an impact on the particle size. As TFR was increased, the particle size was seen to decrease, signifying that the mixing efficiency was increased when more pressure was applied. This can be supported by Zheng *et al.*, where liposome sizes were found to be inversely correlated to TFR at certain FRR and microfluidic devices. They detected that size reduction occurred when the TFR increased, and concluded that this was due to the higher mixing efficiency that can be obtained at higher TFR (Zheng et al., 2022). While the total volumetric flow rate is comparable in this study, the linear flow velocity calculated by dividing the TFR by the cross-sectional area of the flow path is 156 cm/s in SHM and 3 cm/s in SM at TFR 1500 μL/min. Because linear flow velocity is a critical factor in mixing efficiency and shear stress during flow in a path, the significant difference in the linear flow velocity may be involved in the difference in particle size between liposomes formed in SHM and SM. A higher TFR than 2,500 μL/min would be considered for SM to increase the linear velocity when the target size of liposomes is smaller than 150 nm. The impact of FRR on particle size was minor in the range of 3–5. Cheung et al. reported that increasing the FRR resulted in a decrease in liposome size by using the SHM (Cheung and Al-Jamal, 2019). In their study, the range of FRR that affects particle size was between 1 and 3, and the effect on particle size was minor at FRR 3 or above. Therefore, FRR can be a controlling factor for particle size when the FRR is less than 3. At lower FRR, it takes longer for lipids to diffuse into the aqueous phase, allowing the self-assembled intermediate structure known as bilayered phospholipid fragments (BPFs) to grow larger in size, followed by the formation of larger liposomes (Lasic and Martin, 1990; Gouda et al., 2021).

EE was also seen to increase with ILC in both devices. This is likely due to the increase in particle size and number of liposomes, allowing there to be more space in the particles for calcein to be encapsulated. However, when comparing EE to TEE, it can be seen that EE is lower than TEE in both devices. This may be due to the liposomes formulated by SHM, along with SM, not abiding by the assumptions for the TEE model. The model proposed by Xu *et al.* assumes that the liposomes are single lipid bilayers, that the inner and outer aqueous phases have the same concentration, and that the particle size follows a Log-Normal distribution (Xu et al., 2012). Firstly, the liposome particle size may not have followed the Log-Normal distribution, resulting in variation in the TEE and EE. With the particle size changing with the various conditions and devices that were experimented on, it is difficult to confirm that the particle size conformed with the Log-Normal distribution, resulting in the EE differing from the TEE. In addition, the inner aqueous phase may not have the same calcein concentration as the outer aqueous phase, due to potential ethanol being entrapped. When the rate of liposome formation is more rapid than the mixing rate of the two liquids, the calcein solution is not able to be fully mixed with the lipid ethanol solution before forming liposomes, resulting in the encapsulated calcein solution having a lower concentration. In other words, the potential volume for calcein encapsulation is not fully maximized due to some amount of ethanol being entrapped, decreasing the EE. This point seems to be a common issue in both devices, but the differences between EE and TEE were more distinct in SM than in SHM (Supplementary Figure S4). Similarly, the EE was significantly lower than the TEE for the batch-type conventional ethanol injection method (Supplementary Tables S1, S2; Supplementary Figure S7). The significant difference between EE and TEE in SM can be attributed to the lamellar structure of the liposomes. Specifically, the increase in the concentration of lipids combined with the larger particle sizes allows for the liposomes to form multiple layers (Supplementary Figure S5), resulting in lowered EE. Due to the liposomes being more multilamellar, the liposomes formulated with SM have a smaller volume for calcein encapsulation. It has been reported that the encapsulation efficiency of water-soluble fluorescent markers and hydrophilic drugs is considerably low with the conventional ethanol injection method when lipid ethanol solution is injected into the aqueous solutions of the water-soluble molecules (Pons et al., 1993). Although increasing the lipid concentration in the mixed solution is expected to enhance the total encapsulation volume, it also results in the formation of multilamellar or multivesicular liposomes. Also, the BPF and liposome formation may be initiated at higher critical ethanol concentrations at higher ILC. The encapsulation efficiency of these liposomes would be significantly lower than the theoretical encapsulation efficiency calculated assuming unilamellar liposomes encapsulating a completely mixed solution. This issue poses a dilemma when applying the methods based on ethanol injection for the passive encapsulation of water-soluble drugs and may necessitate additional processing to improve encapsulation efficiency. On the other hand, the ethanol injection method demonstrates the efficient encapsulation of hydrophobic and amphiphilic drugs (Jaafar-Maalej et al., 2010; Gouda et al., 2021). Also, electrostatic interactions between encapsulation molecules and lipids, such as a combination of anionic nucleic acids and cationic lipids, are effective in improving encapsulation efficiency (Yang et al., 2012).

In our experimental conditions, SHM was superior to formulate size-controlled unilamellar liposomes around 100 nm independent of the ILC. The unilamellar structure is suitable for the encapsulation of water-soluble molecules, but the low encapsulation efficiency was recognized as a technical issue in this study. On the other hand, SM was characterized by the ability to control the size of liposomes with smaller PDI. In addition, the lamellarity of liposomes is controllable depending on the ILC. The multilayer or multivesicular structure of liposomes formulated by SM at high ILCs may be suitable for stably and efficiently encapsulating hydrophobic drugs and mRNA rather than encapsulation of water-soluble molecules. The structure of the micromixers should be a critical factor to control the lamellarity of liposomes as well as their size. The SHM has an optimized structure for rapid mixing with a herringbone groove in a narrow flow path (Chen et al., 2012). The large flow velocity due to the narrow path allows rapid mixing to form small BPFs after the two liquids were contacted. On the other hand, it can be estimated that the interval between the merging of the two liquids and mixing is extended in SM due to the low flow velocity, giving the lipids time to stay and form larger multilamellar or multivesicular liposomes at the junction of the two liquids. This estimation can be supported by the experiments of preparing lipid nanoparticles using SHMs with different distances between the merging point of liquids and the first SHM, in which the size of the generated lipid nanoparticle is increased by increasing the distance between the merging point and the first SHM (Maeki et al., 2015). The rapid mixing to decrease the ethanol concentration to its critical concentration is crucial to make small-size lipid self-assemblies. They assumed that the formation of BPFs begins at the 80% ethanol condition (mixing rate: 20%) and the BPFs transfer to lipid nanoparticles at the 60% ethanol condition (mixing rate: 40%) (Maeki et al., 2017). The prolonged residence of lipids in a mixing state with 60%–80% ethanol concentrations increases the size of BPFs, which transform into larger lipid nanoparticles when the ethanol concentration drops below 60%. Therefore, control of the residence time of lipids at an ethanol concentration of 60%–80% and the lipid concentration at the critical ethanol concentration can be considered as a key point to control the size of lipid assemblies.

Temperature is also one of the important processing parameters to control the molecular assembling phenomenon because temperature changes the free energy, diffusion coefficient, and viscosity of the systems. In particular, the temperature should be considered as a critical factor when the liposomes exhibit gel-to-liquid crystalline phase transition at a critical temperature. The previous study indicates that liposomes formed at temperatures below the phase transition temperature of the bilayer membranes have larger sizes compared with liposomes prepared around phase transition temperature (Zook and Vreeland, 2010). A further problem in liposome preparation below phase transition temperature is the formation of large aggregates of lipids around the merging zone of liquids, especially at a low flow rate. The formation of lipid aggregates possibly causes the formation of large liposomes with high polydispersity and low reproducibility due to destabilizing the flow. Although the current study was conducted at room temperature, the formation of large aggregates was not observed in either device. This would be due to the addition of an equimolar amount of cholesterol to DPPC, which eliminates the phase transition of the DPPC membrane forming a fluid membrane. Even for liposomes with cholesterol, the heating of the device and fluids increases the diffusion coefficient and decreases the viscosity, resulting in increased mixing efficiency and reduced back pressure. Therefore, microfluidic mixing at higher temperatures allows for rapid mixing to generate smaller liposomes (Cheung and Al-Jamal, 2019).

The element of SM is well-designed for efficient mixing through a process of division, conversion, and inversion (Thakur et al., 2003). The size of multilamellar liposomes can be controlled by the fine mixing process during subsequent passing through the element. Therefore, in addition to the structure of the elements of the static mixer, the design of the channel structure of the micromixer will also be a future issue. The length of the microchannel is one of the considerable parameters in flow mixing. An extension of the microchannel flow path is effective to increase high mixing efficiency to attain a complete mixing state (Natsuhara et al., 2022). However, the back pressure is proportionally increased with increasing the length of the microchannel in pressure-driven flow. Increased back pressure causes breakage of the device. On the other hand, the back pressure is inversely proportional to the square of the channel diameter. In this sense, devices with shorter channels with larger diameters are preferred for safe operation at low back pressure in scale-up processing. SHM has the advantage of being efficiently mixed even in a short channel due to chaotic mixing compared with mixing by hydrodynamic flow focusing through a plane microchannel. However, it is still difficult to increase volumetric throughput because the mixing efficiency decreases due to a deceased specific surface area in the flow channel in which the herringbone grooves are arranged for chaotic mixing when the cross-sectional area of the channel is increased. As a workaround for this limitation in scale-up, a parallelized device with many microchannels has been proposed for the scalable production of mRNA and siRNA lipid nanoparticles (Shepherd et al., 2021). SM can be expected to overcome the limitation of the conventional microfluidic devices in scale-up because the specification of elements of a static mixer such as shape, size, pitch, and length can be customized depending on the length and diameter of the channels.

In addition to statistically visible differences between the two devices, there is a significant difference in the structure and usability of the devices. SHM is a glass material, with the only access to the interior structure being liquids being pushed in through the entrances. Consequently, washing the interior of the device is extremely troublesome with no guarantee that the inside is completely cleansed. On the other hand, as can be seen in Figure 1, SM can be disassembled, allowing for the washing of the device to be done with ease. In addition, by taking out the elements from the device, the elements can be thoroughly washed and experiments can be conducted at virgin state.

## 5 Conclusion

The present study demonstrated that a static mixer is a powerful device for the continuous generation of size-controlled liposomes in a static flow-mixing manner. SM is interesting as a new fluid dynamic mixing device that bridges microfluidic and macro fluidic scales. Similar to the method using conventional microfluidic devices, particle size, encapsulation efficiency, and lamellarity of liposomes could be tuned by modulating process parameters. The key parameter to control the size of liposomes using SM was ILC, which is the same as the method using SHM. TFR and FRR had no significant impact on the characteristics of the generated liposomes in the range tested in this study. However, considering that the cross-sectional area of the channel in the applied SM is 50 times larger than that in the conventional SHM, the range of TFR applied in this study (1,500–2,500 μL/min) might be too low for the SM even if the range of TFR is suitable for SHM in conventional studies (Kastner et al., 2015; Cheung and Al-Jamal, 2019). A much higher TFR range should be targeted to increase the linear flow velocity in the SM. Increased volumetric throughput resulting from an increased TFR is the preferred approach to overcome the limitation of conventional microfluidic devices, which have low volumetric throughput, for scale-up production. SM could be widely applicable not only to preparing liposome formulations but also to preparing nano-formulations based on the formation of molecular assemblies in aqueous media, such as lipid nanoparticles and polymeric nanoparticles. Further research on optimizing manufacturing conditions such as flow rate and temperature to suit the size of the flow path and specifications of SM will enable more precise control of liposome size and structure at various scales.

In the last few decades, liposome technology has played a pioneering role in the development of nanoparticle-based drug delivery systems. While extensive research has led to clinical applications of several liposomal formulations, the number of approved liposomal drugs is not as large as expected. One of the bottleneck issues is manufacturing, which includes inconsistency of size and structure, lack of reproducibility, low encapsulation efficiency, difficulty in scalability, lack of device and equipment, and complexity of the technology. Although there remains a need to better the mixing efficiency of SM by, for instance, altering the number and curvature of the elements, this research was able to show that a static mixer is indeed capable of preparing liposomes. Furthermore, it depicted great potential in liposome formation due to its user-friendliness and ability to accurately control the particle size of the liposomes. Nowadays, liposome technologies have expanded to a broad range of lipid-based nanoparticles including lipid nanoparticles encapsulating DNA or mRNA. Consequently, the challenges of liposome technology extend beyond drug delivery to gene delivery and vaccine technology. Advances in fluid mixing technology using a static mixer will facilitate reproducible, scalable, and efficient continuous production of advanced lipid-based nanoparticles with controlled size and structure suitable for applications.

## Data Availability

The original contributions presented in the study are included in the article/Supplementary Material, further inquiries can be directed to the corresponding author.
