# Supplementary material for: Evaluation of a static mixer as a new microfluidic method for liposome formulation
Source: Front Bioeng Biotechnol. 2023 Aug 22;11:1229829. doi: 10.3389/fbioe.2023.1229829 (PMC10478574; doi:10.3389/fbioe.2023.1229829)
Supplement: Supplementary file 1 [file DataSheet1.PDF]

Supplementary Material

Evaluation of a Static Mixer as a New Microfluidic Method for Liposome Formulation

Aoba Ota, Ayaka Mochizuki, Keitaro Sou, Shinji Takeoka*

*** Correspondence:** Shinji Takeoka: takeoka@waseda.jp

Supplementary Figures

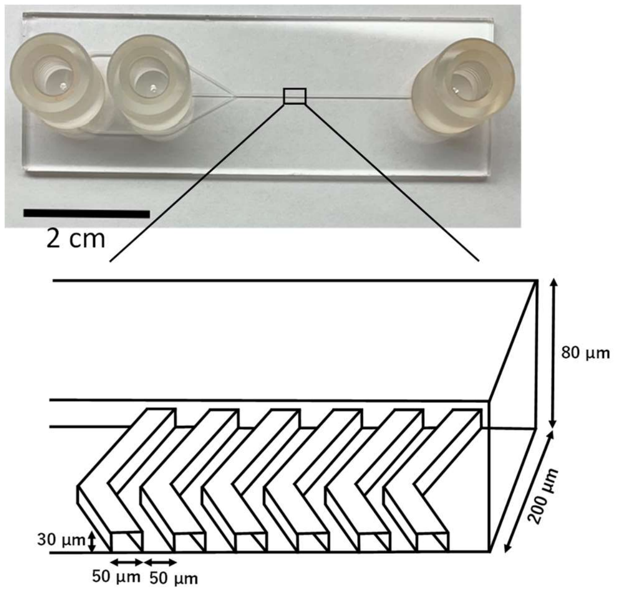

Figure S1. Pictures and three-dimensional structure of the staggered herringbone micromixer (SHM) device.

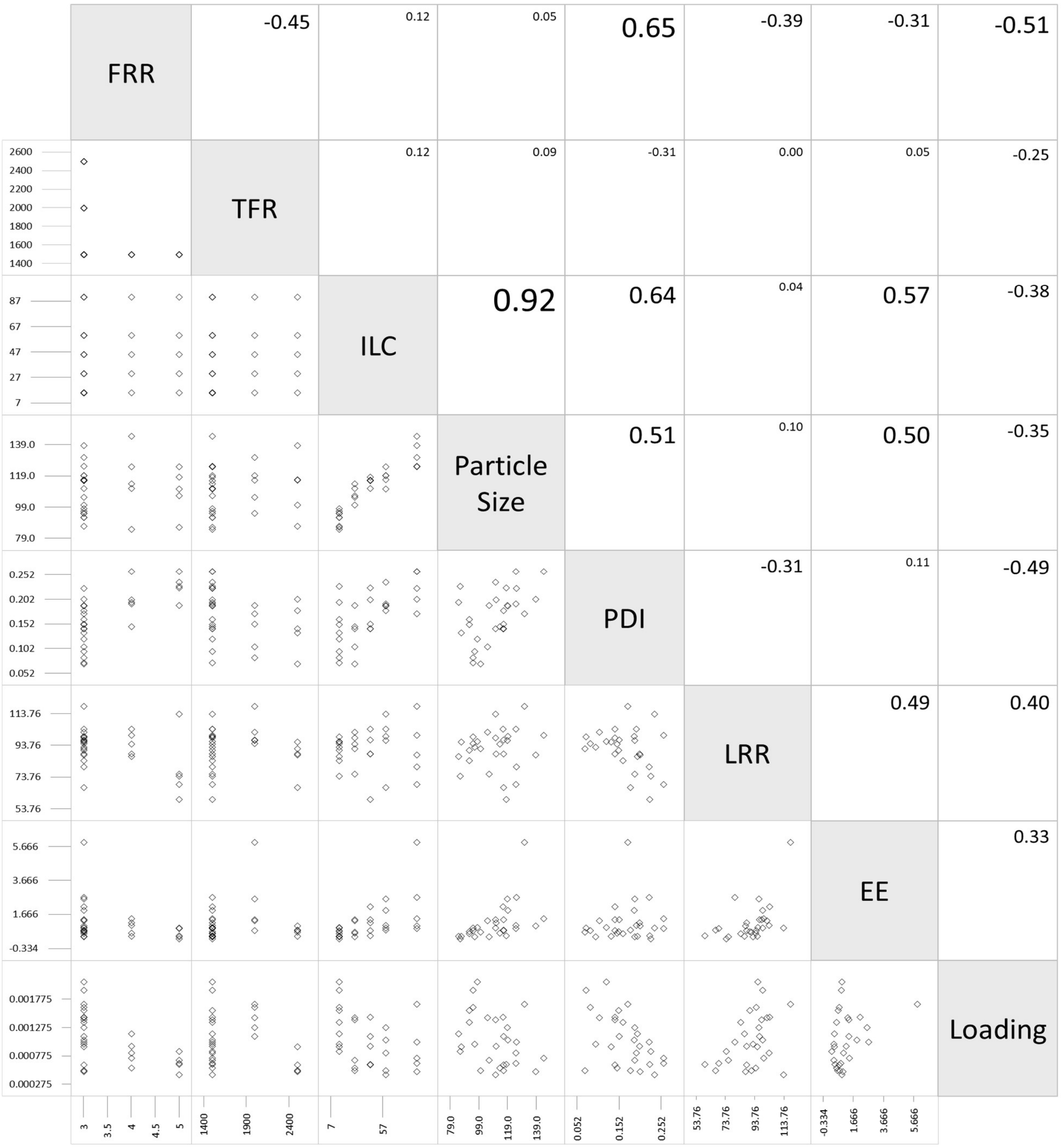

Figure S2. Scatterplot matrix of flow rate ratio (FRR), total flow rate (TFR), initial lipid concentration (ILC), particle size, polydispersity index (PDI), lipid recovery rate (LRR), encapsulation efficiency (EE), and loading for the staggered herringbone micromixer (SHM) device. The values in the boxes represent the Pearson correlation coefficients between each pair of variables.

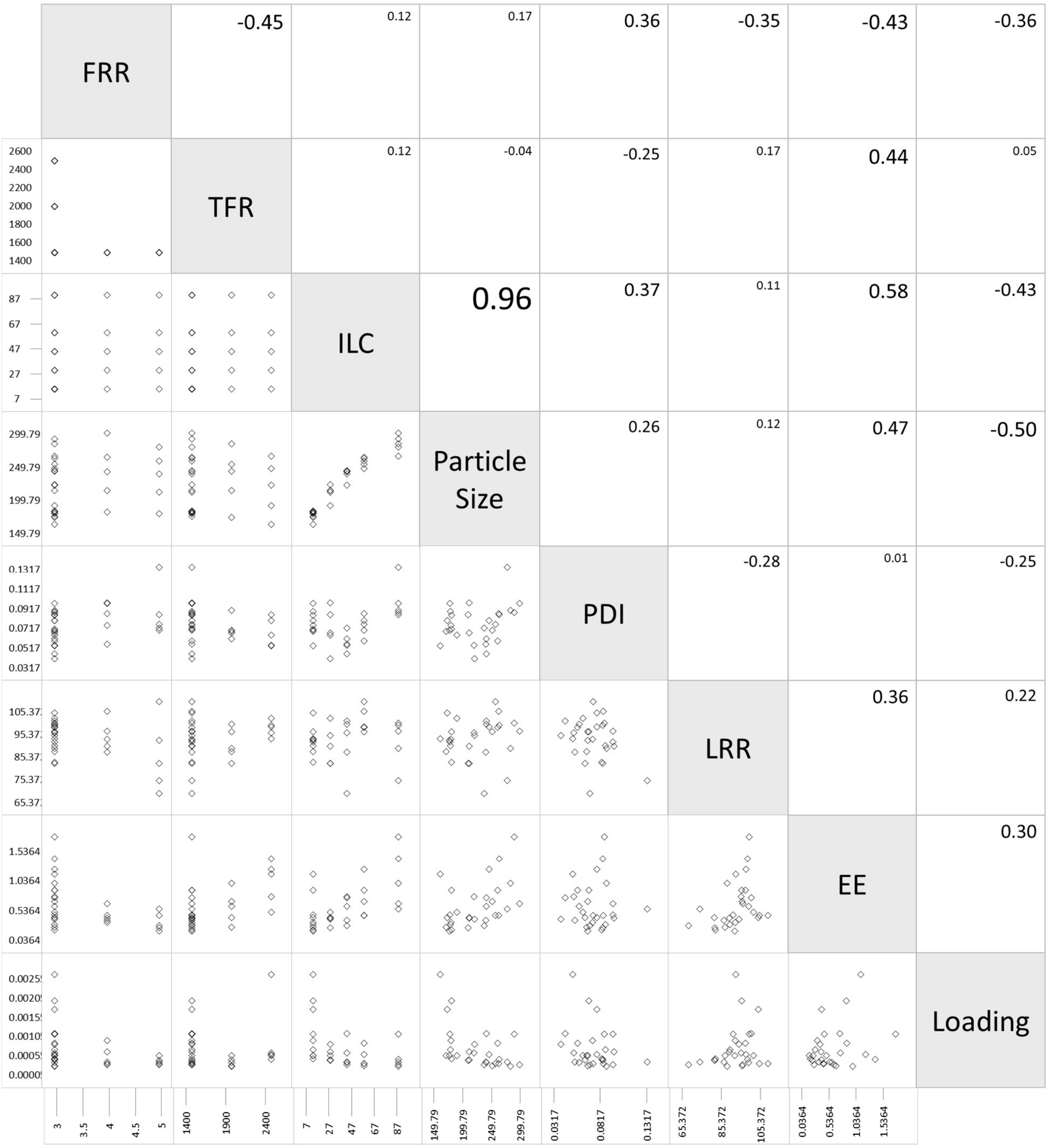

Figure S3. Scatterplot matrix of flow rate ratio (FRR), total flow rate (TFR), initial lipid concentration (ILC), particle size, polydispersity index (PDI), lipid recovery rate (LRR), encapsulation efficiency (EE), and loading for the static mixer. The values in the boxes represent the Pearson correlation coefficients between each pair of variables.

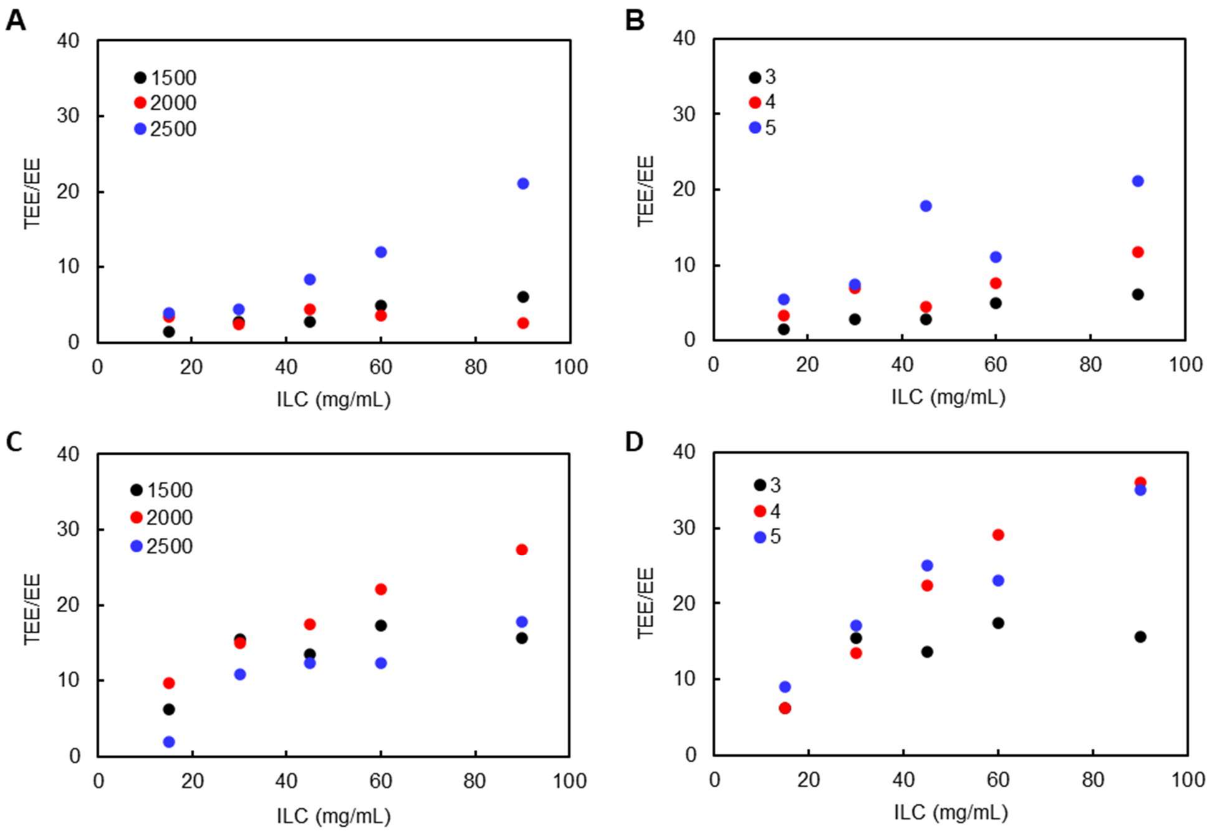

Figure S4. Comparison in the difference between theoretical encapsulation efficiency (TEE) and experimental encapsulation efficiency (EE) of prepared liposomes. (**A**) liposomes prepared with SHM under total flow rate (TFR) 1500, 2000, and 2500, and flow rate ratio (FRR) 3. (**B**) liposomes prepared with SHM under FRR 3, 4, and 5, and TFR 1500, (**C**) liposomes prepared with SM under TFR 1500, 2000, and 2500, and FRR 3, (**D**) liposomes prepared with SM under FRR 3, 4, and 5, and TFR 1500.

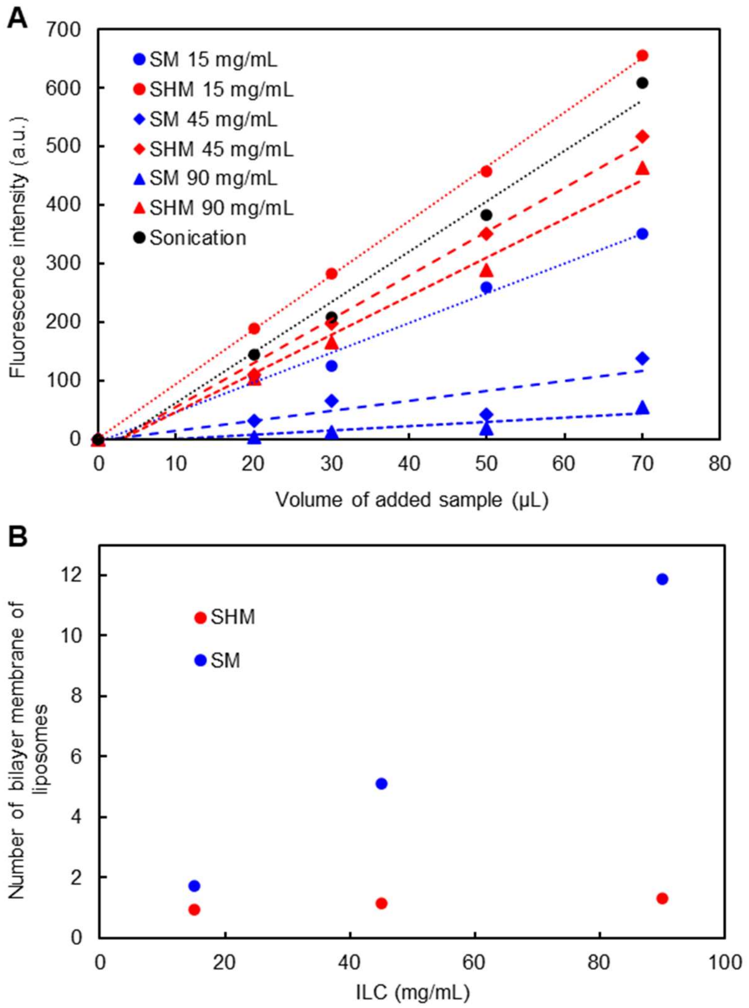

Figure S5. Comparison of the number of bilayer membranes of liposomes formulated by SHM and SM under flow rate ratio (FRR) 3 and total flow rate (TFR) 1500 at initial lipid concentration (ILC) 15, 45, and 90 mg/mL. (**A**) The plots of added volume of liposome samples and fluorescence intensity of 6-p-toluidino-2-naphthalenesulfonic acid (TNS), (**B**) The number of bilayer membrane of liposomes (lamellarity) calculated by the slopes in (**A**). Liposomes prepared by sonication were applied as a standard for unilamellar liposomes.

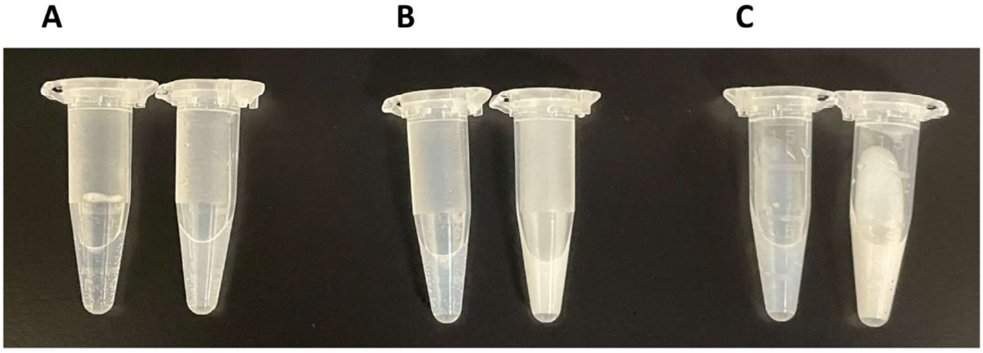

Figure S6. Comparison of the SHM (left) and SM (right) liposome samples at initial lipid concentration (ILC) of (**A**) 15 mg/mL, (**B**) 45 mg/mL, (**C**) 90 mg/mL.

Table S1 Liposomes prepared by conventional ethanol injection method.

ILC (mg/mL)	Mixing volume ratio (aqueous/ethanol)	FLC (mg/mL)	Particle size (nm)	PDI	EE (%)	TEE (%)	TEE/EE	Loading $\times 10^3$
40	12.3	3.0	121±49	0.16	0.28	1.77	6.3	0.76

ILC; initial lipid concentration, FLC; final lipid concentration, PDI; polydispersity index, EE; encapsulation efficiency, TEE; theoretical encapsulation efficiency.

Table S2 Liposome preparation by batch-type ethanol injection method under comparable conditions with experiments using the microfluidic device at FRR 3 and TFR1500.

ILC (mg/mL)	Mixing volume ratio (aqueous/ethanol)	FLC (mg/mL)	Particle size (nm)	PDI	EE (%)	TEE (%)	TEE/EE	Loading $\times 10^3$
15	3	3.8	119±51	0.19	0.58	2.31	4.0	1.39
30	3	7.5	150±55	0.12	1.19	4.96	4.2	1.37
45	3	11.3	172±49	0.08	1.48	7.74	5.2	1.17
60	3	15.0	174±58	0.11	1.91	11.39	6.0	1.10
90	3	22.5	187±68	0.13	2.02	17.09	8.5	0.73

ILC; initial lipid concentration, FLC; final lipid concentration, PDI; polydispersity index, EE; encapsulation efficiency, TEE; theoretical encapsulation efficiency.

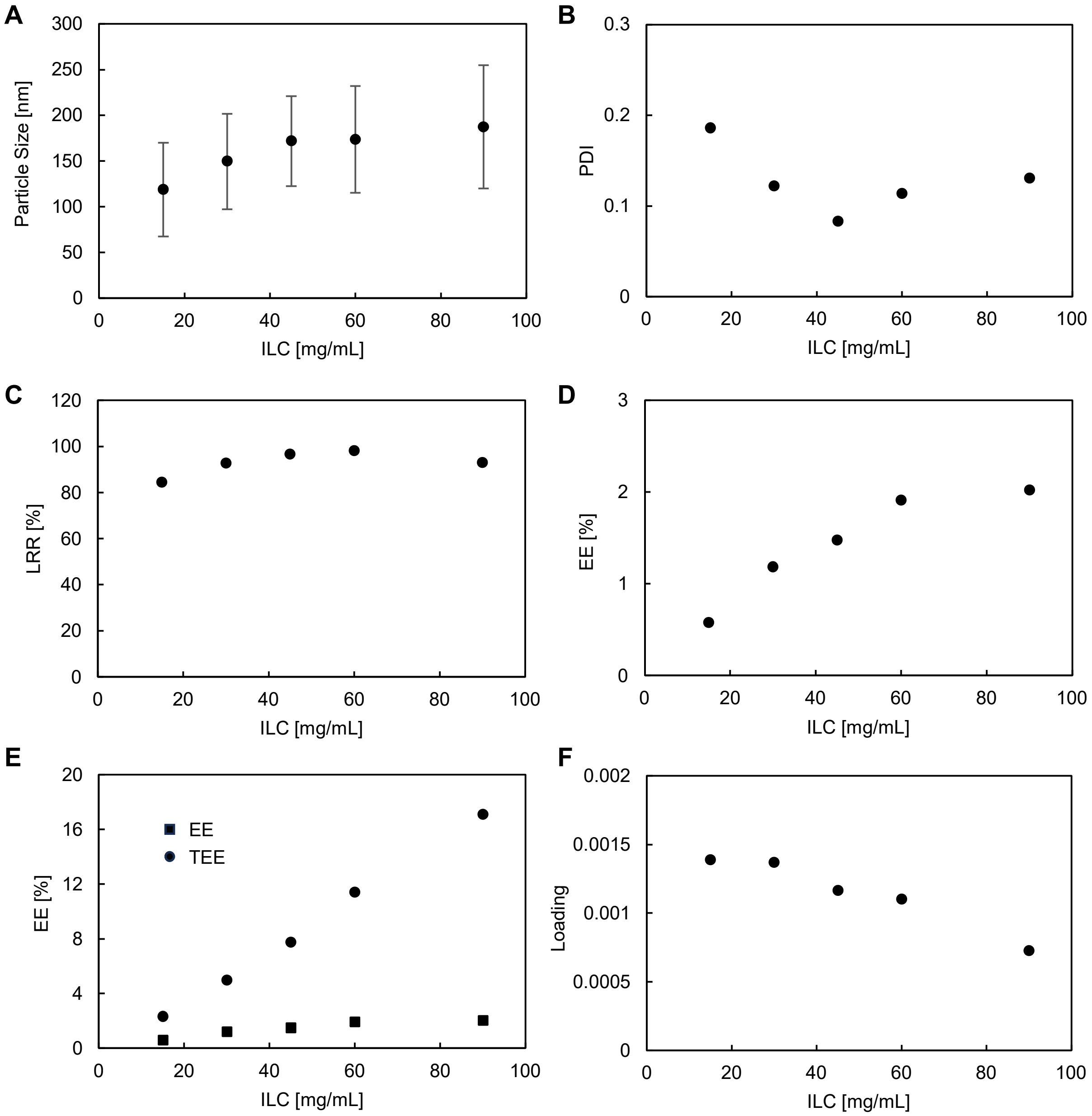

Figure S7. Characteristics of liposomes prepared by batch-type ethanol injection method under comparable conditions with experiments using the microfluidic device at FRR 3 and TFR1500. (**A**) Particle size, (**B**) polydispersity index (PDI), (**C**) lipid recovery rate (LRR), (**D**) encapsulation efficiency (EE), (**E**) theoretical encapsulation efficiency (TEE) and EE, and (**F**) loading.
